# The impact of atopic dermatitis on caregivers’ quality of life in Ethiopia

**DOI:** 10.3389/fmed.2025.1537089

**Published:** 2025-03-12

**Authors:** Abraham Getachew Kelbore, Wendemagegn Enbiale, Jacqueline M. van Wyk, Anisa Mosam

**Affiliations:** ^1^Department of Dermatology, College of Health Sciences and Medicine, Wolaita Sodo University, Wolaita Sodo, Ethiopia; ^2^Department of Dermatology, Nelson R. Mandela School of Medicine, University of KwaZulu-Natal, Durban, South Africa; ^3^Department of Dermatology, College of Health Sciences and Medicine, Bahir Dar University, Bahir Dar, Ethiopia; ^4^Collaborative Research and Training Center for Neglected Tropical Diseases, College of Medicine and Health Sciences, Arba Minch University, Arba Minch, Ethiopia; ^5^Department of Clinical and Professional Practice, Nelson R. Mandela School of Medicine, University of KwaZulu-Natal, Durban, South Africa; ^6^Department of Health Sciences Education, Faculty of Health Sciences, University of Cape Town, Cape Town, South Africa; ^7^Inkosi Albert Luthuli Central Hospital, Durban, South Africa

**Keywords:** eczema, pediatric, family, burden of care, hospital-based study, Ethiopia

## Abstract

**Background:**

Atopic dermatitis (AD) significantly impacts both the physical and psychological well-being of children and caregivers. As AD severity increases, so does its negative effect on the family’s emotional, social, and economic quality. However, the psychosocial and financial challenges faced by caregivers, are often underreported, particularly in developing countries.

**Objectives:**

The study aimed to assess the impact of AD on the quality of life (QoL) of caregivers of children with AD in central and southern Ethiopia.

**Methods:**

A hospital-based cross sectional study was conducted among 461 caregivers of children with AD, from four randomly selected hospitals in Central and Southern Ethiopia between October 2022 and December 2023. A systematic sampling technique was used to enrol study participant Sociodemographic and clinical data were collected by trained nurses. The Dermatitis Family Impact (DFI) questionnaire to assess QoL and the SCORAD index to measure the severity of the diseases. Descriptive statistics, Spearman rank correlation, and one-way analysis of variance (ANOVA) were used for data analysis, with *p*-value < 0.05 considered statistically significant.

**Results:**

Out of 461 AD-diagnosed children, 212 (46%) were girls, and 249 (54%) were boys. The mean DFI score was 9.64 (± 6.44), with 32.3% presenting with mild AD, 46.2% being moderate, and 21.5% with severe AD. The primary caregivers were mostly first-degree family members, with 62% being mothers and 27.2% fathers. A significant correlation was found between the DFI score and the SCORAD index (*p* < 0.0001). The components of quality of life that were adversely affected included sleep, leisure activities, food preparation, emotional distress, tiredness of the caregiver, involvement in treatment, and family relationships. The DFI score was influenced by family occupation, parental education, and comorbidity in children with AD.

**Conclusion:**

Caring for a child with AD adversely affects caregivers or family QoL, which further declines as disease severity increases. This underscores the need for targeted support for caregivers, including practical care management and educational resources, to improve both child and family outcomes.

## Introduction

Atopic dermatitis (AD) is a chronic, relapsing inflammatory skin disease that starts during childhood. It is strongly associated with a family and personal history of atopic diseases, such as asthma, allergic rhinitis, and atopic dermatitis. Childhood AD impacts the quality of life of children and their families (1). Besides genetic susceptibility, environmental and lifestyle factors play a role in developing atopic diseases (2).

Globally, the prevalence of AD varies significantly, ranging from 15% to 20% among children and 1%–6.3% among adults (2–5). Overall, the prevalence of AD in African and Middle Eastern countries has generally been lower than in Europe and North America. However, recent trends show increasing AD prevalence in the developing countries (6). The broadest global epidemiological data sourced from the International Study of Asthma and Allergies in Childhood (ISAAC), estimated the lifetime prevalence of AD in 6–7 years-old children to be relatively low within the eastern Mediterranean and high in Africa (7.2% and 23.3%, respectively) (7, 8). The worldwide prevalence for 6–7 years-old children was 14.2%, and the prevalence of AD in 13–14 years-olds is deemed to be moderate within the eastern Mediterranean (10.8%) and Africa (15.2%) (7). The worldwide prevalence for 13–14 years-old children was 12.8%. The prevalence of AD among children varies in several African countries, Nigeria and Ethiopia, (respectively 7.8% and 9.6%) (9, 10).

Atopic dermatitis has a severe effect on patients and families, impacting the quality of life (QoL) and the social, academic, occupational, and financial aspects of their lives, which accounts for the most considerable global burden of disability owing to skin diseases (11, 12). However, most studies reporting on quality of life (QOL) in AD have come from tools developed in Western cultures, with very few documented studies from other ethnic groups (13, 14). The psychological well-being of the caregiver, with the burden of raising a child with a chronic condition like AD, has been poorly documented in African populations (15, 16). Parents and caregivers of AD children have reported social difficulties and feelings of worry, guilt, blame, and frustration due to their child’s skin disorder (6, 17). The unmeasured expenses on the family unit will undoubtedly be related to the impact of the disease on the parents’ work. Time taken off from work due to doctor visits, poor sleep, depression, and anxiety affect work productivity, salary, and bonuses and may prevent sufficient progress at work. Parents of affected children often stagnate in their careers due to the focus on their child’s needs.

Studies conducted in Western European, and Asian countries confirmed that a greater AD severity negatively affects the quality of life of caregivers/parents (16, 18, 19). They were associated with a more significant negative impact on family life’s physical, emotional, social, and economic components (20).

A study conducted in South Africa revealed that the QOL of caregivers declines with the severity of AD. Factors associated with poorer QOL were: lack of understanding of the disease prognosis, the severity of AD, caring for AD-affected children, the emotional distress of the caregivers, family relationships, and leisure activities (15). Assessing the impact on QoL of caregivers of AD children and its predictors is essential to support and optimize the psychological health of parents and, in so doing, translate into better care for children with AD. Due to a lack of published data, the current study assessed the effect on caregiver QoL among children with AD in the Central and Southern regional states of Ethiopia.

## Materials and methods

### Study setting

The study was conducted in selected dermatology service-providing hospitals in two regions representing Central and South Ethiopia, previously known as part of Southern Nations, Nationalities, and Peoples Regional State (SNNPRS) part of Ethiopia. Central Ethiopia is administratively divided into seven zones and three special districts, while South Ethiopia is divided into 12 Zones (21, 22). This area reportedly includes 76 governmental hospitals, 723 health centers, and 3,874 health posts (23). There are less than 180 dermatology professionals nationwide, and the public healthcare facilities are organized into three-tier healthcare systems: primary (primary healthcare units), secondary, and tertiary. In general, only nine hospitals provide dermatology services by 14 Dermatology professionals in these two regions, and the overall dermatology services are neither accessible nor available in most health facilities.

### Specific setting, study design, and period

The hospital based cross-sectional study was conducted between October 2022 and December 2023 in four public hospitals, which include two comprehensive specialized hospitals (Wolaita sodo University and Nigistelen Mohammed Memorial) and two general hospitals (Arbaminch and Dr. Bogalech Gebre Memorial). The hospitals provide services to black skinned people of Ethiopian origin. These hospitals provide holistic dermatology services, including inpatient management of dermatological emergencies.

### Study population

The population of interest included children, aged 16 years or younger diagnosed with AD during the study period and their designated caregiver. Children with a known psychiatric problem, a history of steroid and antihistamine treatment intake within the last 2 weeks, and families or caregivers aged 18 years or less were excluded.

### Sample size determination

For this study, a sample size of (*n* = 470), as calculated as published previously, was used (24). A 95% confidence interval and margin of error (0.03%) was used. Caregivers of the children were enrolled based on the inclusion criteria.

### Sampling technique

Four zones were randomly selected from the two regions. After that, the available hospitals offering dermatological services from the chosen zones were purposively chosen, and the sample size was proportionally allocated to each hospital based on the average number of patients visiting each hospital in the last 6 months. Then, the hospitals were stratified into the ranks of the hospitals (i.e., secondary and tertiary levels). Finally, participants, parents, or caregivers from the selected hospitals were selected through systematic sampling.

### Study variables and measurement

The main research variables were the clinical profile of patients with Atopic dermatitis, age, sex, residence, family occupation, family educational status, marital status of caregiver/family, blood relation with a caregiver, atopy history, onset of age, duration since diagnosed, comorbidity, and gender of caregiver/family, history of, and, and clinical severity of the disease according to SCORAD index.

In this study, United Kingdom working party diagnostic criteria for AD, and validated for use in Ethiopian children (25, 26) were used to ascertain the diagnosis.

Clinical dermatologists assessed the severity using the SCORAD index, which considers both objective (clinical presentation and extent) and subjective AD scores. The numerical data on all three aspects of the disease were entered into tables and summed, with a maximum score of 103. A SCORAD index score of less than 25 indicates mild disease, 25–50 indicates moderate severity and greater than 50 indicates severe AD. Asymptomatic patients have a SCORAD result of 0.

The caregiver who accompanied the child to the hospital was selected for the interview. If multiple individuals accompanied the child, they were asked to designate one representative to participate in the interview.

Caregivers were interviewed using the Dermatitis Family Impact (DFI) questionnaire, a 10-item tool designed specifically for caregivers of children with atopic dermatitis (AD). The DFI assessed how the child’s AD affected daily life and family activities over the past week, including housework, meal preparation, leisure, shopping, medical costs, fatigue, and family relationships. Responses were scored as: “very much” (3 points), “a lot” (2 points), “a little” (1 point), and “not at all” (0 points), with higher scores showing a greater impact. The total score ranged from 0 to 30 with higher scores indicating poorer QoL.

### Operational definition

A caregiver: someone who may or may not have a blood relationship with the child but cares for the sick child at home and anywhere.

### Data collection tool

A structured interview questionnaire was created based on a literature review and adjusted to suit the local context. The tools were translated from English to Amharic and back to English by different professional translators to ensure accuracy. All children with AD were examined by trained dermatologists, and data collectors (nurses) received training on the tools, procedures, and informed consent process before starting. Data collection took place at the dermatology outpatient units of the hospitals.

### Data processing and analysis

Collected data was double-entered into Epi-Data software *(v4.2.0.0 for entry Epi-Data Association, Odense, Denmark)*. The data was cleaned by running frequency and cross-tabulation using Epi-data and then exported to SPSS version 27 for analysis. Descriptive statistics, including frequencies, the proportion at (95% CI), mean, standard deviation, and median, were calculated. The association between the DFI score and AD severity (SCORAD index score) was described according to a Pearson correlation coefficient. Spearman rank correlation was used to describe the association between the DFI score and the number of dependents. The difference in DFI scores between independent variables was assessed using a one-way analysis of variance (ANOVA). Associations between DFI scores and categorical variables were investigated using the *Post-hoc* test. Results with a *p*-value < 0.05 were considered statistically significant. The Cronbach’s alpha statistic was used to measure the internal consistency of the DFI questionnaire.

### Data quality assurance

To maintain the quality of data, a pre-validated standard questionnaire was used for data collection. Continuous supervision was done during data collection at each hospital, and ten percent of the data set was double-entered to check the data’s accuracy.

## Results

### Characteristics of the study population

Four hundred sixty-one caregivers of AD patients who met the inclusion criteria were recruited. The median age was 3 years, the Interquartile range (IQR) was 1,5), and the largest proportion of children were less than 5 years old, 312 (67.7%). The age of caregivers ranged from 18 to 54 years (mean 30.43 ± 5.84 years). Three hundred fifteen (68.3%) of caregivers were female, and four hundred eight (88.5%) of caregivers were married. According SCORAD, 149 (32.3%) patients were mild, 213 (46.2%), moderate and 99 (21.5%) had severe AD.

In this study, the first-degree family was the primary caregiver with 94.8%; of these, the mother was the primary caregiver in 286 (62%) of cases. Three hundred fifty-eight caregivers (77.7%) had at least one other dependent younger than 16 years of age living with them. Table 1 describes the sociodemographic characteristics of caregivers and patients.

**TABLE 1:** Sociodemographic characteristics of the caregivers and children with atopic dermatitis in central and Southern Ethiopia 2023/2024.

Variables	Category	Frequency	Percent
Sex	Female	212	46
	Male	249	54
Age of children	≤ 4 years	312	67.7
	5–10 years	102	22.1
	11–16 years	47	10.2
Residence	Rural	174	37.7
	Urban	287	62.3
Family occupation	Employee	185	40.1
	Farmer	59	12.8
	Housewife	77	17.7
	Merchant	140	30.4
Education status mother	No formal education	102	22.1
	Primary school	99	21.5
	Secondary school	75	16.3
	Tertiary (diploma and above)	185	40.1
Education status father	No formal education	79	17.1
	Primary school	82	17.8
	Secondary school	80	17.4
	Tertiary (diploma and above)	220	47.7
Marital status caregiver	Widowed/divorced	23	5
	Married	408	88.5
	Single	30	6.5
Age of caregiver	18–24 years	41	8.9
	25–34 years	295	64
	≥ 35 years	125	27.1
Caregiver gender	Female	315	68.3
	Male	146	31.7
Number of children ≤ 16 yrs	< 2	103	22.3
	≥ 2	358	77.7
How long since diagnosed	< 1 year	310	67.2
	≥ 1 year	151	32.8
Blood relation with caregiver	Mother	286	62
	Father	125	27.1
	Siblings	26	5.6
	Other	24	5.2
Comorbidity of child	No	362	78.5
	Yes	99	21.5
Comorbid diseases of child	Asthma	71	71.7
	Diabetes mellitus	19	19.2
	Other	9	9.1
Comorbidity of caregivers	None	321	69.6
	Diabetes mellitus	25	5.4
	HIV/AIDS	3	0.7
	Hypertension	22	4.8
	Asthma	79	17.1
	Other	11	2.4

### Dermatitis Family Impact (DFI) questionnaire

A high Cronbach’s alpha (0.881) suggested good internal consistency of the DFI questionnaire and indicated a reliable scale. A one-way ANOVA was conducted to determine if the scores of DFI were different across AD severity status according to the SCORAD index. The mean DFI and standard deviation (SD) was 9.64 (± 6.44). Participants were grouped into three groups: children with mild AD (*n* = 149), moderate AD (*n* = 213), and severe AD (*n* = 99), according to the SCORAD index. Also, the mean DFI was 6.75 (± 5.22) for mild AD, 9.2 (± 5.76) for moderate AD, and 15 (± 6.3) for severe AD. There was a significant correlation between the mean DFI score and AD severity (*p* < 0.0001).

The DFI score was not impacted by marital status (*p* = 0.98, r = −0.001), number of children below 16 years old in the family (*p* = 0.101, r = 0.077), the caregiver blood relation (*P* = 0.97, r = 0.001) and among caregivers or families who have comorbidities (*p* = 0.953, r = 0.003).

The DFI score differed depending on family occupation (*p* = 0.01, r = 0.2), maternal and paternal educational status (*p* = 0.01, r = 0.18), and children with AD comorbidities (*p* = 0.001, r = 0.12).

**FIGURE 1:**
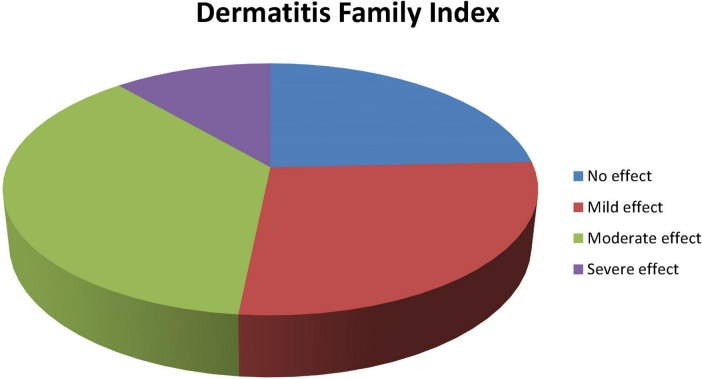
Distribution of impact on quality of life among caregivers of atopic dermatitis (AD) children according to the Dermatitis Family Impact (DFI) scale in selected hospitals in Ethiopia.

Figure 1 shows the distribution of the DFI scale measured among the caregivers of children with AD being moderate in 171 (37.1%) caregivers, by mild in 126 (27.3%), no impact in 112 (24.3%) and severe effect in 52 (11.3%), respectively.

### Housework

Caring for a child with AD had no impact on housework activities such as washing and cleaning in 334 (72.45%) of all cases. “No effect” or “a little” was noted in 104 (69.8%) and 33 (22.1%) of mild cases of AD, respectively. Housework was affected “a lot” or “very much” in moderate AD 8 (3.8%) and Severe AD cases 16 (16.2%).

### Food preparation and feeding

This category referred to purchasing specific foods or ingredients, the child’s dietary restrictions with AD and family members, the extra time and attention needed for meal preparation, and the costs involved. “Little” or “no effect” was reported in 122 (81.9%) of mild cases, whereas the effect was reported as “a lot” in 49 (23%) of moderate cases, and the effect was “very much” was reported in 28 (28.3%).

### Sleep

In severe AD cases, *n* = 43 (43.4%) of caregivers described their sleep as being affected “a lot,” *n* = 33 (33.3%) of caregivers described their sleep as being affected “very much.” Sleep was not affected in *n* = 60 (40.3%) mild AD cases.

### Family leisure activities, shopping, and expenditure

Caregiver’s leisure activities were not affected in 135 (29.3%) of all AD children, and only 46 (9.97%) of caregivers reported a “very much” effect on all aspects of leisure activities. Only 156 (33.8%) of caregivers reported that caring for a child with AD had “a lot” or “very much” of an effect on all aspects of shopping for their family. Costs related to treatment, clothing, etc., were impacted “a lot” or “very much” in 135 (29.3%) of all cases.

### Tiredness and emotional distress of caregivers

Responses to questions on tiredness had a fair representation: 190 (41.2%) of caregivers reported “no tiredness” caused by caring for a child with AD, 137 (29.7%) described the effect as “a little,” *n* = 92 (19.96%) as “a lot” and *n* = 42 (9.1%) as “very much” of all cases of AD. Caregivers described feelings such as depression, frustration, or guilt in 281 (61%) of all cases.

### Relationships

The caregiver’s relationship with their partner and other children was affected “a little” in *n* = 100 (46.9%) moderate cases and *n* = 58 (38.9%) mild cases. A total of 152 (82.15%) caregivers reported that caring for a child with AD affects their relationship with their partner and other children “a lot” or “very much” in all cases.

### Helping with treatment

Helping with the child’s treatment affected the caregiver’s life in 352 (76.4%) AD cases. The effect was experienced as “a lot” in *n* = 40 (40.4%) and “very much” in *n* = 25 (25.3%) of the severe cases. Caregivers reported “no effect” in *n* = 50 (33.6%) of mild cases and *n* = 49 (23%) in moderate cases of AD (Table 2).

**TABLE 2:** Dermatitis Family Impact scores across different SCOAD index categories in atopic dermatitis children (*N* = 461), central and Southern Ethiopia 2023/2024.

Category	Effect	Mild (*N* = 149), *n* (%)	Moderate (*N* = 213), *n* (%)	Severe (*N* = 99), *n* (%)	*P*-value
Housework	Not at all	104 (69.8)	162 (76.1)	68 (68.7)	0.815
	A little	33 (22.1)	43 (20.2)	15 (15.2)	–
	A lot	8 (5.4)	7 (3.3)	5 (5.1)	–
	Very much	4 (2.7)	1 (0.5)	11 (11.1)	–
Food preparation	Not at all	81 (54.4)	58 (27.2)	8 (8.1)	0.001
	A little	41 (27.5)	97 (45.5)	19 (19.2)	–
	A lot	18 (12.1)	49 (23)	44 (44.4)	–
	Very much	9 (6%)	9 (4.2)	28 (28.3)	–
Sleep	Not at all	60 (40.3)	44 (20.7)	5 (5.1)	0.0001
	A little	54 (36.2)	86 (40.4)	18 (18.2)	–
	A lot	27 (18.1)	61 (28.6)	43 (43.4)	–
	Very much	8 (5.4)	22 (10.3)	33 (33.3)	–
Leisure	Not at all	72 (48.3)	56 (26.8)	7 (7.1)	0.0001
	A little	48 (32.2)	92 (43.2)	21 (21.2)	–
	A lot	24 (16.1)	50 (23.50)	45 (45.5)	–
	Very much	5 (3.4)	15 (7)	26 (26.3)	–
Shopping	Not at all	127 (85.2)	175 (82.2)	79 (79.8)	0.219
	A little	11 (7.4)	21 (9.9)	4 (4)	–
	A lot	3 (2)	13 (6.1)	10 (10.1)	–
	Very much	8 (5.4)	4 (1.9)	6 (6.1)	–
Expenditure	Not at all	84 (56.4)	83 (39)	25 (25.3)	0.0001
	A little	43 (28.9)	79 (37.1)	12 (12.1)	–
	A lot	17 (11.4)	43 (20.2)	41 (41.4)	–
	Very much	5 (3.4)	8 (3.8)	21 (21.2)	–
Tiredness	Not at all	82 (55)	79 (37.1)	29 (29.3)	0.0001
	A little	41 (27.5)	76 (35.7)	20 (20.2)	–
	A lot	21 (14.1)	40 (18.8)	31 (31.2)	–
	Very much	5 (3.4)	18 (8.5)	19 (19.2)	–
Distress	Not at all	81 (54.4)	72 (33.8)	27 (27.3)	0.0001
	A little	43 (28.9)	73 (34.3)	27 (27.3)	–
	A lot	17 (11.4)	51 (23.9)	28 (28.3)	–
	Very much	8 (5.4)	17 (8)	17 (17.2)	–
Relationship	Not at all	69 (46.3)	53 (24.9)	7 (7.1)	0.0001
	A little	58 (38.9)	100 (46.9)	22 (22.2)	–
	A lot	21 (14.1)	47 (22.1)	39 (39.4)	–
	Very much	1 (0.7)	13 (6.1)	31 (31.3)	–
Treatment	Not at all	50 (33.6)	49 (23)	10 (10.1)	0.0001
	A little	47 (31.5)	97 (45.5)	24 (24.2)	–
	A lot	40 (26.8)	53 (24.9)	40 (40.4)	–
	Very much	12 (8.1)	14 (6.6)	25 (25.3)	–

*P*-value < 0.05 determined as statistically significant.

### Correlation between the SCORAD index and the Dermatitis Family Impact questionnaire

The overall responses to the DFI questions were significantly associated (*p* < 0.05) with the SCORAD index, except for housework (*p* = 0.81, r = 0.011) and shopping (*p* = 0.22, r = 0.06) (Table 2).

**FIGURE 2:**
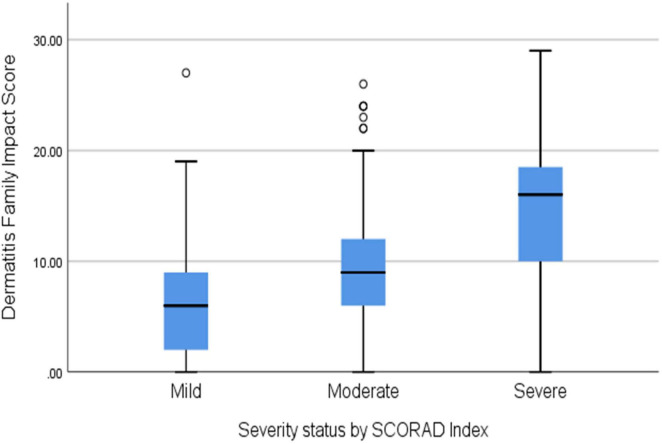
The distribution of Dermatitis Family Index scores across the category of atopic dermatitis (AD) severity by the SCORAD index (*p* < 0.0001).

Quality of life factors significantly affected with moderate to low correlation were the caregiver emotional distress of the caregiver (*p* < 0.000, r = 0.32), tiredness of the caregiver (*p* < 0.0001, r = 0.33), family leisure activities (*p* < 0.0001, r = 0.47), expenditure (*p* < 0.0001, r = 0.38), Helping with treatment (*p* = 0.001, r = 0.29), food preparation and feeding (*p* = 0.001, r = 0.49), sleep of family members (*p* = 0.001, r = 0.45), the caregiver’s relationships (*p* = 0.001, r = 0.5). Figure 2 shows the distribution of DFI scores for each category of the Objective SCORAD index.

## Discussion

This study is the first to document the impact of atopic dermatitis (AD) on the quality of life (QoL) of caregivers in Ethiopia. Our findings show that caregivers of children with AD experience significant adverse effects on their QoL, which is strongly correlated with the severity of the disease in the child for whom they care.

In our study, the median age of the children was 3 years, with 67.7% being under 5 years old. Younger children typically require more caregiving for daily activities, which may contribute to the increased caregiving burden. The primary caregivers in our study were mainly first-degree family members (94.8%), including mothers (62%), fathers (27.2%), and siblings (5.6%), with more than two-thirds being female caregivers. This emphasizes the significant responsibility placed on mothers, who are often the primary caregivers.

Our findings indicate that over 80% of caregivers of children with severe AD reported significant disruptions in their QoL. This is consistent with the work of Neri et al. (27), which found that parents often struggled at work due to poor sleep, were forced to change their working hours, or even changed jobs due to the burden of caregiving. Similarly, Chamlin et al. (28), reported that many parents resort to co-sleeping to prevent scratching, which leads to disrupted sleep patterns and additional emotional stress. This practice may affect the caregivers’ sleep quality and lead to habitual behavior in children, even when the disease is under control.

Our study found that 82% of caregivers reported that caring for a child with AD affected their relationship with their partner and other children, leading to social isolation and strained family dynamics. This is consistent with findings from South Africa and other Western countries (29–31), where AD is associated with social isolation (32). Although impaired parental sleep is often thought to be directly related to the child’s night time awakenings, adjusting for sleep disturbances in our study did not change the results. Other studies suggest that parental sleep disturbances are linked to psychological distress and depression, which are prevalent in caregivers of children with AD (33). In Ethiopia, many families have limited sleeping facilities and often share beds, which could further contribute to the disruption of sleep and caregiving stress (34). This study did not assess these environmental factors, but they should be considered in future research.

The impact on family leisure activities, such as recreation, swimming, and holidays, was moderate to severe for caregivers of children with moderate and severe AD (30.7% and 51.8%, respectively). Our findings align with previous studies that reported restrictions on leisure activities due to AD (35), such as avoiding swimming in pools and exposure to hard water that exacerbates the condition (36, 37). These restrictions may be compounded by social, cultural, and economic factors in Ethiopia, which influence how families spend their leisure time.

In terms of financial burden, our study found that expenditures related to treatment, clothing, and other AD care items had a significant impact on caregivers. Approximately 29.3% of caregivers rated the financial burden as “a lot” or “very much,” which is consistent with findings from Barbarot et al. (20), who reported that severe AD significantly impacted the family’s physical, emotional, social, and economic aspects. While some studies from South Africa suggest that child support grants may help alleviate the financial burden, this was not statistically associated with AD severity (15). However, caring for a child with moderate or severe AD had a higher financial burden than caring for one with insulin-dependent diabetes mellitus (15, 38). Socioeconomic factors, health infrastructure, and the family’s understanding of the disease may explain these differences.

Interestingly, housework and shopping activities were not significantly correlated with AD severity in our study, but practical caregiving tasks such as applying topical treatments and cleaning soiled clothes due to weeping skin were mentioned as sources of stress for caregivers. These tasks are often not captured in traditional assessments but add to the daily strain of caregiving (39).

Food preparation was another significant factor impacting caregivers, particularly as some families followed specific diets to manage the condition (40, 41). While some studies suggest a link between diet and AD management, the evidence is inconclusive (40, 42). Caregivers may face additional costs and stress due to dietary restrictions, which can increase the burden of managing AD (40). Educating caregivers about food allergies and the role of diet in AD management could potentially alleviate some of this burden (43).

Our study also found a strong correlation between disease severity (measured using the SCORAD index) and caregiver QoL, as assessed by the Dermatitis Family Impact (DFI) questionnaire. Previous studies have also found a positive correlation between AD severity and the impact on caregivers’ QoL Moreover, the DFI score varied depending on factors such as family occupation, parental educational status, and the presence of comorbidities in the children. This suggests that socioeconomic factors play a significant role in the caregiving burden.

Caring for children with AD needs family time investment, which could affect the family work absence and the family’s level of education, which matters in the awareness of disease care and follow-up. However, the DFI score does not vary across marital status, the number of dependent children in the family, and the DFI score for caregivers or families who have comorbidities. This is a consistent finding with a study from South Africa (15).

Additionally, our study found that caregivers’ cost of treatment of a child with AD had a notable impact on their quality of life in the majority of cases, AD causes a considerable economic burden on families with costs related to treatment and indirect expenses (32, 44). Caregivers may have worries relating to medication use, particularly topical steroids (6, 17). Some studies pointed out the importance of educational programs on AD that might positively impact the course of the disease and the family’s quality of life by providing psychological support to the patients and their parents. Training of the caregiver and the patients with AD has a significant influence on the therapeutic success and treatment adherence of patients’ with AD (45).

Our study was conducted at a dermatology clinic and a possible limitation linked to the comprehensive specialized and general hospital setting may be a bias toward more severe cases as reflected by the higher proportion of moderate and severe AD cases in our sample. Further studies using more invasive yet accurate methods, such as tape stripping, could assess AD severity, identify associated cutaneous biomarkers, track therapeutic responses in pediatric AD, and predict future disease progression and comorbidities (46).

## Conclusion

In conclusion, caring for a child with AD severely impacts caregivers’ QoL, with the burden increasing with disease severity. Sleep disturbances, financial strain, family relationships, expenditure, emotional well-being and involvement in treatment are all significantly affected by AD severity. Addressing the medical and psychosocial aspects of AD, providing support services, and raising awareness about the disease is crucial for improving the QoL of caregivers. Healthcare providers should offer resources, guidance, and emotional support to caregivers to help mitigate these impacts.

## Data Availability

The raw data supporting the conclusions of this article will be made available by the authors, without undue reservation.
